# Differential contributions of the middle frontal gyrus functional connectivity to literacy and numeracy

**DOI:** 10.1038/s41598-017-17702-6

**Published:** 2017-12-13

**Authors:** Maki S. Koyama, David O’Connor, Zarrar Shehzad, Michael P. Milham

**Affiliations:** 1 Nathan Kline Institute for Psychiatric Research, Orangeburg, NY USA; 2 Haskins Laboratories, New Haven, CT USA; 3 Child Mind Institute, New York, NY USA; 4 Yale University, Department of Psychology, New Haven, CT USA

## Abstract

Literacy and numeracy equally affect an individual’s success in and beyond schools, but these two competencies tend to be separately examined, particularly in neuroimaging studies. The current resting-state fMRI study examined the neural correlates of literacy and numeracy in the same sample of healthy adults. We first used an exploratory “Multivariate Distance Matrix Regression” (MDMR) approach to examine intrinsic functional connectivity (iFC), highlighting the middle frontal gyrus (MFG) for both competencies. Notably, there was a hemispheric asymmetry in the MDMR-based MFG findings, with literacy associated with the left MFG, whereas numeracy associated with the right MFG (R.MFG). Results of post-hoc seed-based correlation analyses further strengthened differential contributions of MFG connections to each competency. One of the most striking and novel findings from the present work was that numeracy was negatively related to R.MFG connections with the default network, which has been largely overlooked in the literature. Our results are largely consistent with prior neuroimaging work showing distinct neural mechanisms underlying literacy and numeracy, and also indicate potentially common iFC profiles to both competencies (e.g., R.MFG with cerebellum). Taken together, our iFC findings have a potential to provide novel insights into neural bases of literacy, numeracy, and impairments in these competencies.

## Introduction

Literacy and numeracy are relatively modern inventions in the course of human evolution. Acquisition of these achievement skills involves explicit learning of abstract symbols (e.g., letters, digits, mathematical symbols) and rules, as well as their repeated application, in and beyond schools. Both literacy and numeracy directly impact the academic and financial success of an individual throughout his or her life^1^. This is made obvious by the increased rates of negative outcomes in individuals affected by learning disabilities (LD), such as academic dropout, unemployment, and imprisonment^2^. Not surprisingly, an expansive literature examining behavioral and neural correlates of literacy and numeracy has emerged^3–8^. Unfortunately, these competencies tend to be examined in isolation of one another (i.e., not being examined in the same population), with studies more commonly focusing on literacy and LD with literacy (dyslexia) than numeracy and LD with numeracy (dyscalculia). This is clearly reflected in a higher number of publications for dyslexia than dyscalculia^9^.

In large part, the tendency for neuroimaging studies to examine literacy and numeracy in isolation of one another can be attributed to an assumption that distinct processes underlie each of the competencies. For example, dyslexia is commonly associated with deficits in phonological awareness^10,11^, while dyscalculia is often characterized by deficits in numerical magnitude processing^12,13^. Supporting the notion that distinct deficits underlying each of these LDs, a review of literature on previous structural and functional MRI studies has implicated differential patterns of connectivity and activation for literacy and numeracy^7^. Specifically, those of literacy have implicated a distributed array of regions in the left hemisphere, including the prefrontal, temporo-parietal, and occipito-temporal regions^4,6,14^. In contrast, those of numeracy have emphasized the involvement of bilateral fronto-parietal networks^3,15–17^, as well as the importance of crosstalk between the left and right hemispheres^18^.

Despite various distinctions between literacy and numeracy, there are several commonalities between the two competencies. First, is the relatively high frequency of comorbidity between dyslexia and dyscalculia^19,20^. Second, is the shared need for domain-general cognitive competencies, such as attention^21,22^. This is made evident by findings of attentional deficits in both dyslexia and dyscalculia^23^. Finally, there are overlaps in the abnormalities revealed for the two LDs by fMRI studies. For example, while abnormalities in the fronto-parietal network are frequently cited for dyscalculia^13,17,24^, they are also reported in dyslexia^4,25^, just to a lesser degree. These observations draw attention to potential limitations in the practice of studying literacy and numeracy in isolation of one another.

The present work aimed to examine the neural correlates of literacy and numeracy in the same sample of healthy adults, using the publicly available Nathan Kline Institute-Rockland Sample (NKI-RS)^26^. We focused specifically on resting-state functional MRI (R-fMRI) to examine intrinsic functional connectivity (iFC) of literacy and numeracy. Task-free R-fMRI approaches, which allow researchers to avoid the challenges in designing probe tasks, have successfully been used to study neural correlates of higher cognitive functions, including reading^27–30^, arithmetic^31,32^, and intelligence^33,34^. To relate iFC to literacy and numeracy performance, we first employed Multivariate Distance Matrix Regression (MDMR)^35^, which is an exploratory analysis that attempts to explain inter-individual differences (i.e., distances) in whole-brain iFC profiles of each voxel in terms of one or more phenotypic variables of interest.

Importantly, MDMR represents a recent analytic shift, from univariate (e.g., seed-correlation analysis [SCA]) to multivariate iFC analyses, the latter of which rely minimally on a priori assumptions. Although the majority of previous R-fMRI studies on literacy^25,28^ and numeracy^31^ employed SCA, which can provide a direct answer to a direct question (thus straightforward interpretability), this method may, by definition, fail to identify potentially important iFC patterns of regions that have been rarely reported in the task-evoked fMRI literature. To overcome such potentially biased findings and map iFC underlying literacy and numeracy more comprehensively, we performed both MDMR and post-hoc SCA using MDMR-based findings/clusters that do not specify the direction of connectivity-behavior relationships (i.e., positive or negative)^35–37^.

Specifically, two subtests of the Wechsler Individual Achievement Test (WIAT)^38^, “Word Reading” (literacy) and “Numerical Operations” (numeracy), were the behavioral phenotypic variables of interest in the present study. The WIAT has been one of the most commonly used standardized tests in both educational and clinical evaluations, largely assessing progress and achievement of individuals learning to read/spell and calculate in general classrooms. Both subtests measure individuals’ abilities to retrieve the knowledge (i.e., grapheme-phoneme correspondence for “Word Reading”; arithmetic facts for “Numerical Operations”) and apply it to a given question that is visually presented. We believe that the use of these two WIAT subtests enables us to examine iFC associated with literacy and numeracy achievements in adults.

## Results

### Behavioral Results

**Figure 1:**
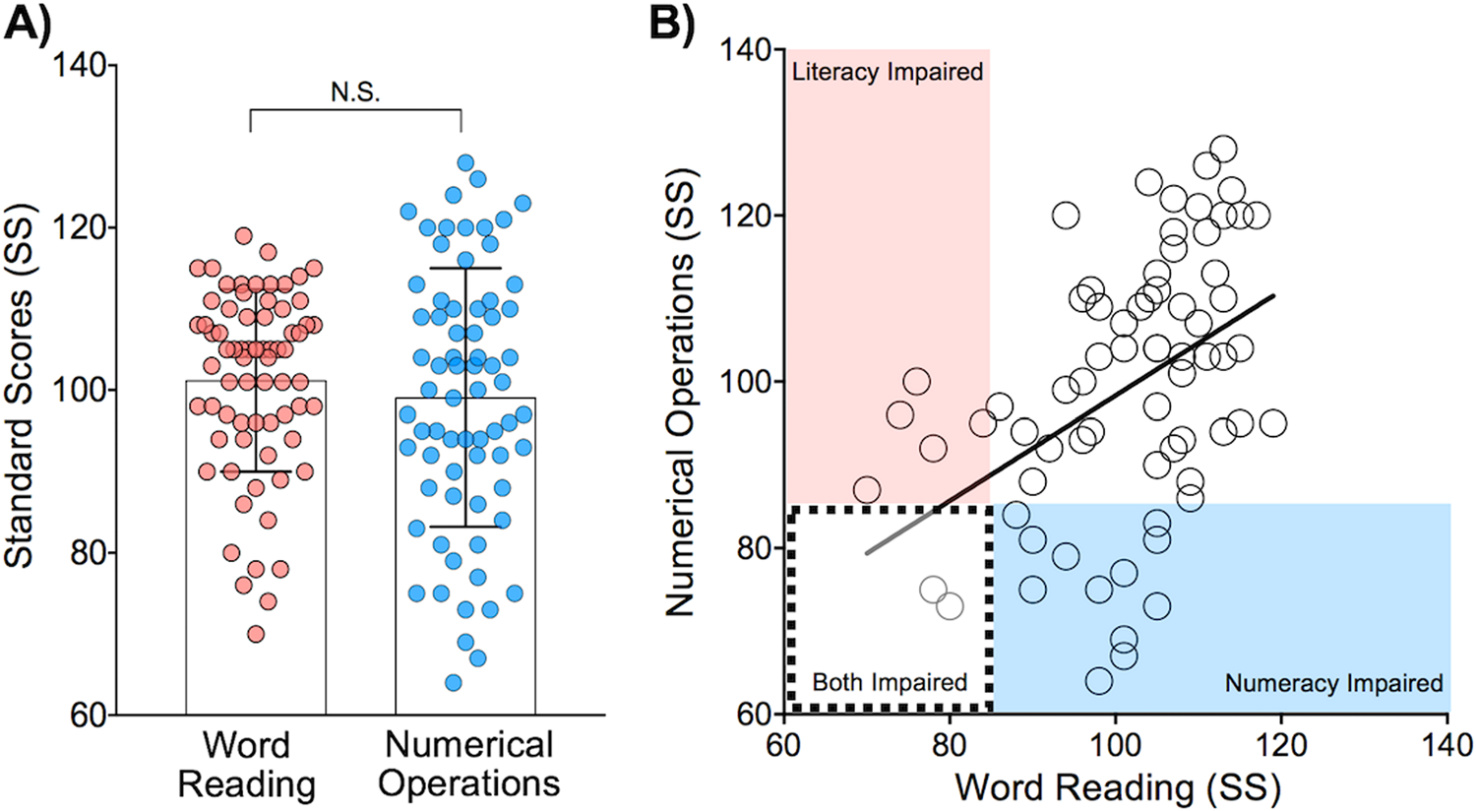
Performance on literacy and numeracy. (**A**) The group mean performance was not significantly different between literacy (Word Reading) and numeracy (Numerical Operations). (**B**) Individuals impaired in literacy are shown in the pink & dashed-line boxes (n = 7, 10%), whereas those impaired in numeracy are in the blue and dashed-line boxes (n = 14, 20%). Among those identified, two individuals impaired in both competencies are shown within the dashed-line box. SS = Standard Scores.

Table 1 summarizes demographic, cognitive, and behavioral profiles of the participants included in statistical analyses (n = 70). None of them had any psychiatric, intellectual, or attentional deficits. Figure 1A shows that the performance (i.e., standard scores) was not significantly different between Word Reading (literacy) and Numerical Operations (numeracy) (paired t-test; *t* = 1.19, *p* = 0.24). As expected^38^, literacy and numeracy were moderately correlated with each other (*r* = 0.44, *p* < 0.01) (Fig. 1B). In our sample, we detected seven individuals with a significant impairment in literacy (10%) (Fig. 1B: circles in the pink and dashed-line boxes), and fourteen individuals with numeracy impairment (20%) (Fig. 1B: circles in the blue and dashed-line boxes). Among those identified, two individuals exhibited impairments in both literacy and numeracy (Fig. 1B: circles in the dashed-line box). Note that “impairment” in the current study was defined as having a standard score lower than 85 (i.e., −1SD). The prevalence estimate for impaired literacy in the current study is consistent with a previous report (5~17.5%)^39^. For numeracy, although the incidence of clinically significant impairments has been reported to be 3~6% in children^40^, a recent large-scale study in UK has revealed more than 20% of adults with poor numeracy skills^41^. This high ratio among adults is consistent with that observed in our sample. These observations suggest that our community-ascertained sample can be considered to be a representative sample.

**Table 1:** Demographic, cognitive, and behavioral profiles of the participants (n = 70).

	Mean	SD	Range
Age (Years)	30.76	10.03	20–49
Sex	26 M: 44 F	—	—
WASI Full Scale IQ	101.30	8.85	84–129
VASI Verbal IQ	98.73	9.95	81–132
WASI Performance IQ	99.89	8.91	85–127
WIAT Word Reading	101.20	11.19	70–119
WIAT Numerical Operations	99.10	15.90	64–128
Edinburgh Handedness	77.36	19.14	50–100
CAARS INA	44.57	7.58	35–66
CAARS HYP/IMP	43.40	6.48	29–62
Mean FD	0.06	0.02	0.03–0.17

SD = Standard Deviation, WASI = Wechsler Abbreviated Scale of Intelligence, WIAT = Wechsler Individual Achievement Test, CAARS = Conners’ Adult ADHD Rating Scales, INA = Inattentiveness, HYP/IMP = Hyperactivity/Impulsivity, FD = Frame-wise Displacement.

### R-fMRI results with exploratory MDMR

**Figure 2:**
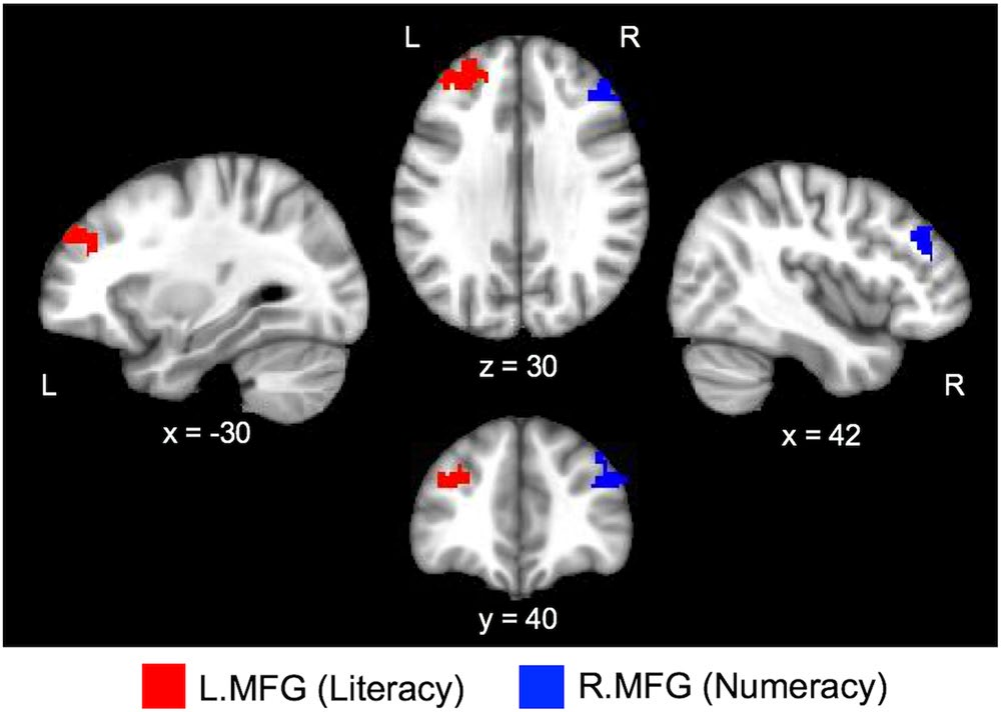
Multivariate distance matrix regression results. Literacy (Word Reading) was associated with the anterior-ventral part of the left middle frontal gyrus (L.MFG in red), whereas numeracy (Numerical Operations) was associated with the anterior-ventral part of the right middle frontal gyrus (R.MFG in dark blue). Cluster-level correction of Z > 3.1, p < 0.05.

MDMR analyses revealed that literacy was associated with the iFC of the anterior-ventral part of the left middle frontal gyrus (L.MFG: the peak MNI coordinates; x = −30, y = 46, z = 33), whereas numeracy was associated with the iFC of the anterior-ventral part of the right middle frontal gyrus (R.MFG: the peak MNI coordinates: x = 42, y = 38, z = 30) (Fig. 2). These two clusters largely consisted of homotopic brain areas (i.e., the same brain areas in opposite hemispheres). We corrected for multiple comparisons using cluster-based extent thresholding, with a height (i.e., cluster-forming) threshold of *Z* > 3.1 (corresponding to *p* < 0.001) and cluster-extent probability of *p* < 0.05 (Family-wise error rate [FWER] corrected), using Gaussian Random Fields (GRF).

### R-fMRI results with post-hoc SCA

Consistent with prior neuroimaging studies using the MDMR framework^35^, we next performed post-hoc SCA for each of the L.MFG and R.MFG clusters, to identify specific connections that may be contributing to the MDMR-based findings. For SCA results, we again applied the *Z* > 3.1, *p* < 0.05, GRF-based correction for multiple comparisons. The SCA results further strengthened differential patterns of iFC for literacy and numeracy, and each result is described below.

**Figure 3:**
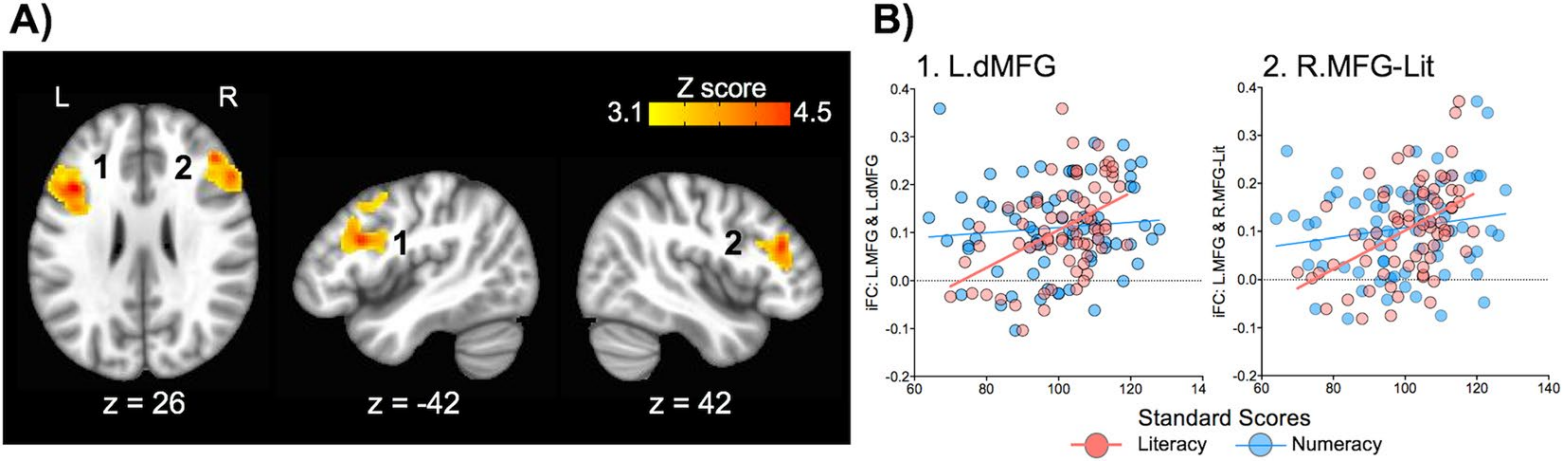
Seed-based correlation analysis (SCA) of the left middle frontal gyrus (L.MFG) for its positive relationships with literacy. (**A**) Literacy (Word Reading) was positively associated with two L.MFG connections with 1) the dorsal part of the left middle frontal gyrus (L.dMFG), extending into the left inferior frontal gyrus and 2) the right middle frontal gyrus (R.MFG-Lit). (**B**) Scatter plots show that these L.MFG connections were significantly associated only with literacy, but not with numeracy. Corrected for cluster-level of Z > 3.1, p < 0.05. L = Left, R = Right.

**Figure 4:**
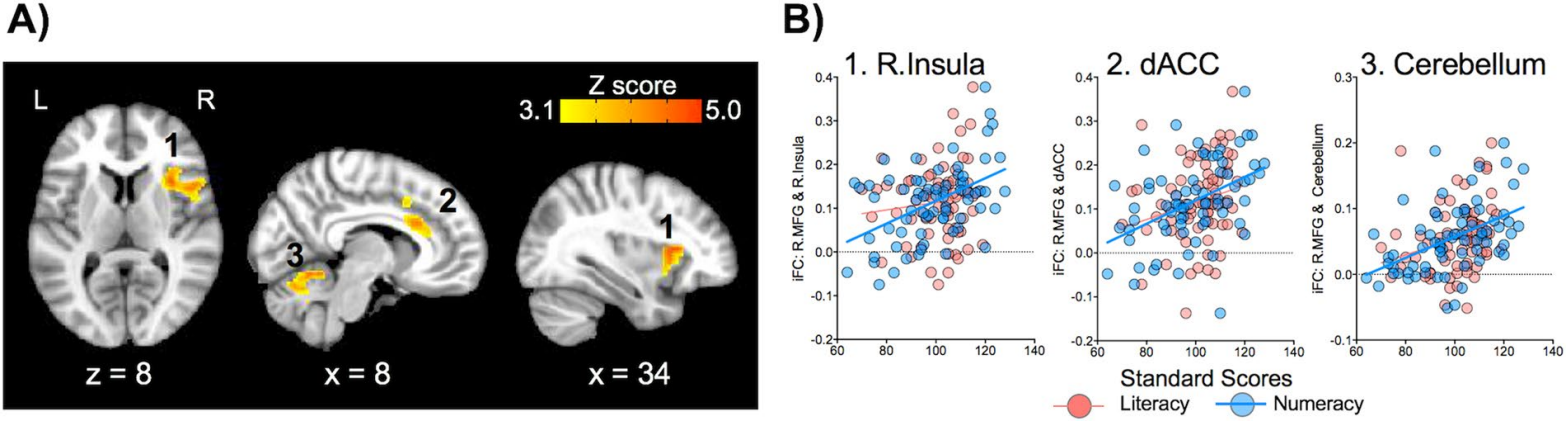
Seed-based correlation analysis (SCA) of the right middle frontal gyrus (R.MFG) for its positive relationships with numeracy. (**A**) Numeracy (Numerical Operations) was positively associated with three R.MFG connections with; (1) the right insula (R.Insula), (2) the dorsal part of the anterior cingulate cortex (dACC), and (3) the cerebellum (Cereb). (**B**) Scatter plots show that these connections, except for R.Insula, were significantly associated with not only numeracy but also literacy. Corrected for cluster-level of Z > 3.1, p < 0.05. L = Left, R = Right.

As shown in Fig. 3A, literacy was positively related to iFC between L.MFG and two clusters: 1) the dorsal part of L.MFG, extending into the left inferior frontal gyrus (L.dMFG, the peak MNI coordinates: x = −42, y = 18, z = 26), located dorsally to the L.MFG seed, and 2) the right middle frontal gyrus (R.MFG-Lit, the peak MNI coordinates: x = 44, y = 34, z = 20), located immediately inferior and posterior to the MDMR-based R.MFG finding associated with numeracy (nearly no overlap between these two R.MFG clusters). No negative connectivity-behavior relationship was observed for literacy. For numeracy, the performance was positively associated with three R.MFG connections (Fig. 4A): 1) the anterior part of the right insula (R.Insula: the peak MNI coordinates: x = 34, y = 18, z = 8), 2) the dorsal part of the anterior cingulate cortex (dACC: the peak MNI coordinates: x = 8, y = 18, z = 26), and 3) the bilateral cerebellum (Cereb: the peak MNI coordinates: x = 10, y = −50, z = −12), including lobules IV, V, VI, and VII. Of note, the insula and cingulate areas identified are located within the salience network^42,43^.

**Figure 5:**
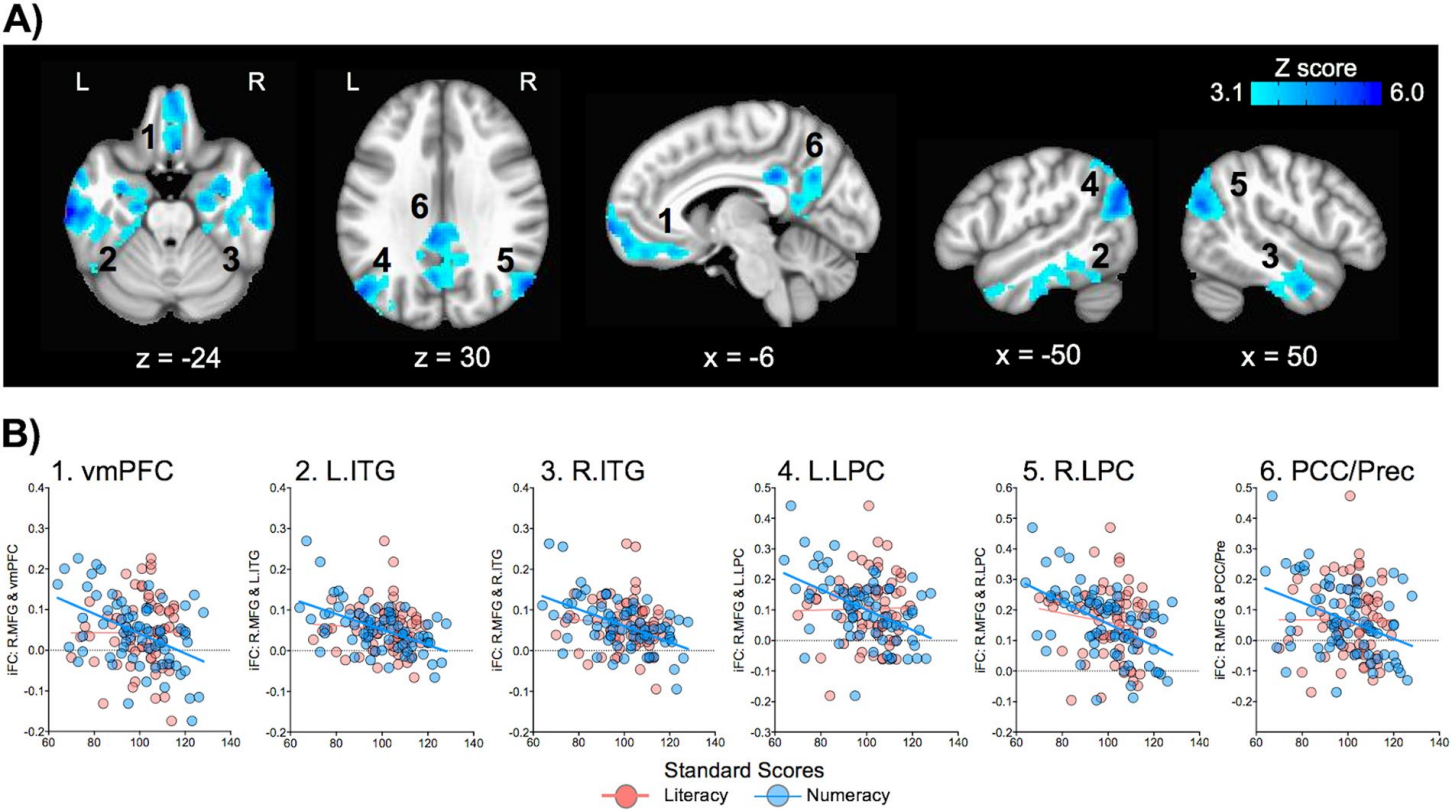
Seed-based correlation analysis (SCA) of the right middle frontal gyrus (R.MFG) for its negative relationships with numeracy. (**A**) Numeracy (Numerical Operations) was negatively associated with six R.MFG connections with; 1) the ventromedial prefrontal cortex (vmPFC), 2) the left inferior temporal gyrus (L.ITG), 3) the right anterior ITG (R.ITG), 4) the left lateral parietal cortex (L.LPC), 5) the right LPC (R.LPC), and 6) posterior cingulate cortex/precuneus (PCC/Prec). (**B**) Scatter plots show that these connections were significantly associated with numeracy, but not with literacy. Corrected for cluster-level of Z < 3.1, p < 0.05. L = Left, R = Right.

Numeracy was also negatively associated with iFC between R.MFG and six clusters, all of which appeared to be located within the default network^44,45^. As shown in Fig. 5A, these included: 1) the ventromedial prefrontal cortex (vmPFC: the peak MNI coordinates: x = 2, y = 52, z = −26), 2) the left inferior temporal gyrus (L.ITG: the peak MNI coordinates: x = −64, y = −20, z = −24), 3) the anterior part of the right inferior temporal gyrus (R.ITG: the peak MNI coordinates: x = 64, y = −4, z = −24), 4) the left lateral parietal cortex (L.LPC: the peak MNI coordinates: x = −50, y = −70, z = 30), 5) the right lateral parietal cortex (R.LPC: the peak MNI coordinates: x = 52, y = −68, z = 28), and 6) the posterior cingulate cortex, extending into the precuneus (PCC/Pre: the peak MNI coordinates: x = −6, y = −38, z = 30). That is, higher numeracy performance was associated with weaker positive (or stronger negative) R.MFG connections with these default network regions. Note that clusters within LPC can overlap with or be adjacent to the intraparietal sulcus (IPS), a core region implicated in numerical^46^ and arithmetic processing^47^. Given that the role of the lateral part of the parietal cortex is prominent in the default network^48^ and that our findings included other default networks regions for numeracy, we consistently used a label of “LPC”, rather than “IPS”.

To examine if the SCA-based findings were specifically associated with either literacy or numeracy, we performed two additional analyses: 1) plotting each of the identified connections as a function of the two competencies, and 2) calculating the difference between two connectivity-behavior correlations^49^ (i.e., one correlation for literacy and anther for numeracy), with one variable in common (i.e., iFC), for each connection. As shown in Fig. 3B, neither of the L.MFG connections identified for literacy was significantly associated with numeracy (neither of the correlations was *p* < 0.05). For each of these L.MFG connections, there was a significant difference between the two connectivity-behavior correlations (both *Z* scores were *p* < 0.05). These results indicate that the L.MFG connections are specific to literacy. For the R.MFG connections identified for numeracy, none of them, except for the dACC (*R* ^2^ *= *0.06, *p* < 0.05) and Cereb (*R* ^2^ = 0.07, *p* < 0.05) clusters, were significantly associated with literacy (Figs 4B and 5B). Similarly, for each of these connections, except for the dACC *(Z* = 1.8, *p* > 0.05) and Cereb (*Z* = 1.9, *p* > 0.05) clusters, the two connectivity-behavior correlations were significantly different. These results indicate that the R.MFG connections with R.Insula and the default network are specific to numeracy, whereas two R.MFG connections with dACC and Cereb may be common iFC profiles to the two competencies.

**Figure 6:**
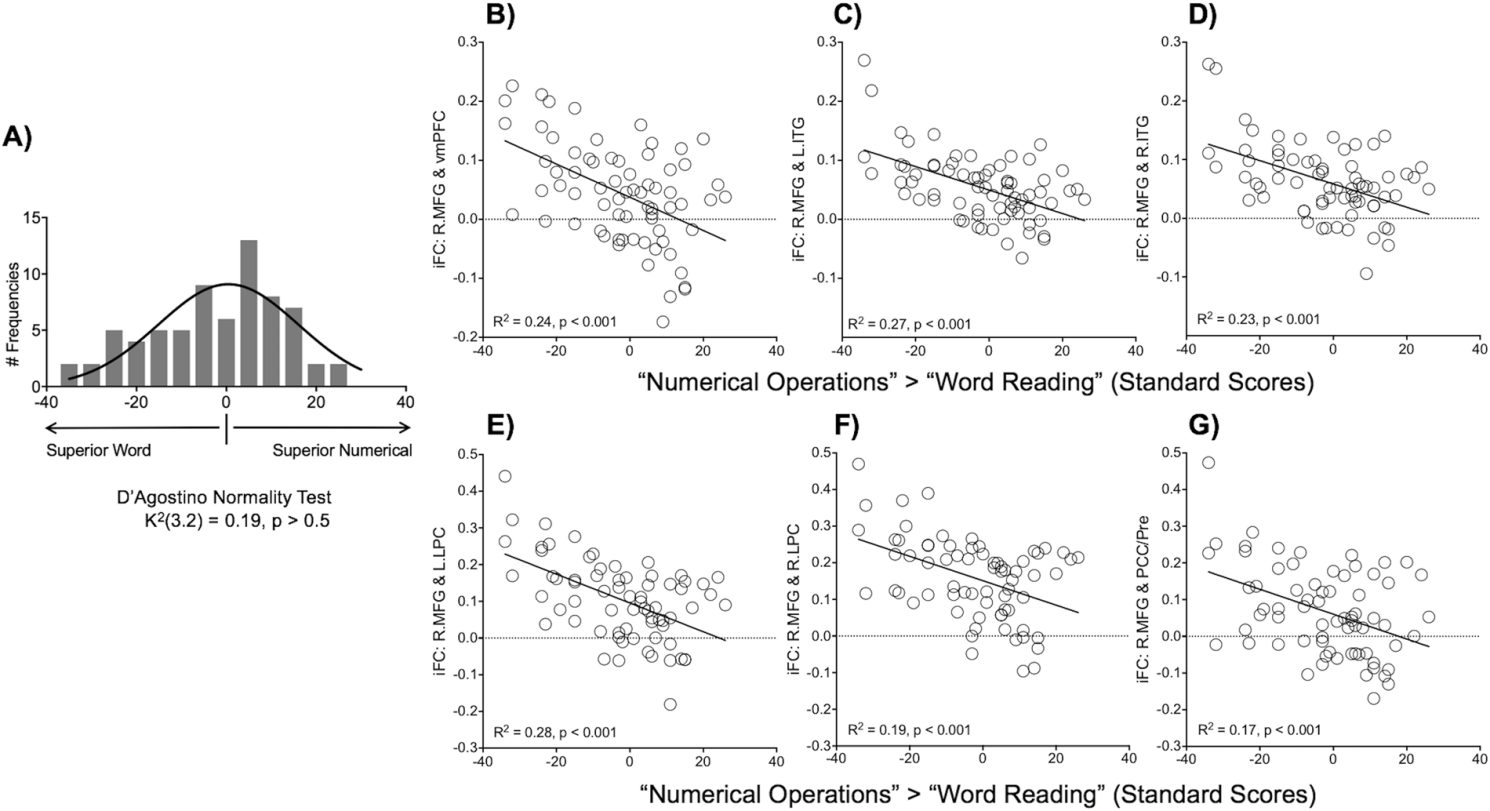
The discrepancy in standard scores between numeracy and literacy. (**A**) Discrepancy scores (“Numerical Operations > Word Reading”) were normally distributed in the sample. (**B)** Scatter plots show that R.MFG connections with default network regions were negatively associated with the discrepancy, that is, the more superior the numeracy to literacy, the weaker the positive iFC (or the stronger the negative iFC). vmPFC = ventromedial prefrontal cortex, L.ITG = left inferior temporal gyrus, R.ITG = right anterior ITG, L.PLC = left lateral parietal cortex, R.LPC = right LPC, PCC/Pre = posterior cingulate cortex/precuneus.

From the additional analyses above, it is evident that all R.MFG connections with default network regions are specific to numeracy. Because the roles of the default network in numeracy remains relatively unknown, we performed a secondary analysis, attempting to identify potential sources of these negative connectivity-behavior relationships. Specifically, we calculated the discrepancy in standard scores between Numerical Operations and Word Reading (i.e., Numerical minus Word), which were then linked to the R.MFG connections with default network regions. To clarify, an individual who performed Numerical Operations more superiorly to Word Reading (“Superior Numerical”) had a positive value for the discrepancy score, and vice versa. First, we confirmed that the discrepancy scores in our sample were normally distributed, tested by D’Agostino test (*p* > 0.05) (Fig. 6A). Second, scatter plots revealed a strikingly consistent pattern across all the connections; individuals with “Superior Numerical” (Numerical Operations > Word Reading) tended to have weaker positive (or stronger negative) iFC in each of these connections (Fig. 6B–G). This pattern coincides with the aforementioned negative connectivity-behavior relationships (i.e., the greater the numeracy, the weaker the positive iFC).

In addition, given that R.MFG connections exhibited both positive (Num +) and negative (Num −) relationships with numeracy, we examined how these two types of R.MFG connections were associated with each other (Supplementary Figure 1A). Connectivity strength between R.MFG and R.Insula (a “Num + connection”) had a significant negative correlation with each of R. MFG connections with the default network regions (“Num – connections”), especially with the parietal clusters (e.g., R.LPC, *r = *−0.58). That is, individuals with stronger positive connectivity strength in R.MFG's “–Num + connection” tended to have weaker positive (or stronger negative) connectivity strength in R.MFG's “Num − connections” (Supplementary Figure 1B), indicating functional segregation between the two opponent network systems. Note that this pattern was not significant for other “Num + connections” (i.e., dACC, Cereb). Additionally, within each of “Num + connections” and “Num − connections”, there were strong positive correlations, indicating functional integration within each of the task-positive and task-negative (default) network systems (Supplementary Figure 1A). These iFC findings, derived from connectivity-behavior relationship analyses, coincide with previous work that delineates concurrent within-network integrations and between-network segregations in the resting brain^50–52^.

## Discussion

Our application of a recently proposed exploratory multivariate analysis framework (MDMR) in analysis of R-fMRI data revealed distinct neural indices of literacy and numeracy. The MDMR findings implicated the middle frontal gyrus (MFG), as a core component of the multiple demand system^53^, for both competencies. However, it is important to emphasize a hemispheric asymmetry in our MFG findings; specifically, literacy was associated with L.MFG, while numeracy was associated with R.MFG. Such a neural dissociation in these two competencies was further strengthened by post-hoc SCA findings, among which R.MFG iFC with the default network was most striking, though largely overlooked in the task-evoked fMRI literature to date (but see^16,54^).

The involvement of MFG in both literacy and numeracy is not surprising given that prior studies implicate this region as a part of the multiple demand system^53,55,56^. Regions in this system tend to exhibit a common pattern of responsivity to an array of cognitive tasks, including word reading (i.e., non-word reading)^56^ and numerical operations (i.e., addition)^56^. For word reading, L.MFG is commonly implicated across different languages, from alphabetic English to logographic Chinese^57^. For numerical cognition, R.MFG has been activated during calculation tasks, particularly those involving working memory manipulations^58–60^. These previous task-evoked fMRI findings are consistent with our MDMR findings, emphasizing differential hemispheric contributions of MFG connections to literacy and numeracy. That is, the whole-brain iFC associated with literacy is left lateralized, contrasted with the right lateralization for numeracy. These lateralization patterns render further support for the well-established left hemisphere predominance for language abilities^61^, including word reading^30,62,63^, and also coincide with prior work showing greater reliance on right hemisphere functions for numerical^64^ and arithmetic^7^ processes. Such right-hemispheric importance in numeracy may reflect the unique non-verbal demands of numerical operations^65–68^, given that R.MFG is specifically activated during learning of non-verbal information (patterns), but not verbal information (words)^69^.

One of the most novel findings in the present work is the specific involvement of the default network in numeracy. Specifically, the R.MFG connections with core default network regions^44,45^ (vmPFC, ITG, LPC, PCC/Pre) were negatively associated with numeracy. That is, individuals with greater functional segregation, defined as stronger negative (or weaker positive) iFC between task-positive prefrontal regions and task-negative default network regions, tended to have better numeracy performance. To date, only a few fMRI studies of numeracy and dyscalculia have reported the default network^54,70^, yet their findings are consistent with our findings. For example, relative to unimpaired controls, children with numeracy impairment exhibited hyper-connectivity (i.e., stronger positive connectivity) between prefrontal and parietal default network regions during a numerical task^47,71^. Similarly, a R-fMRI study has shown that children with numeracy impairment were characterized by atypically increased local intrinsic activity (so-called “fractional amplitude of low frequency fluctuations”^72^) in a distributed array of regions, including R. MFG and a parietal default network region^32^.

Collectively, the previous and current findings indicate a beneficial role of stronger negative (or weaker positive) connectivity between task-positive prefrontal and task-negative default parietal regions in successful numeracy performance. Yet, a crucial question remains as to why such negative connectivity-behavior relationships, anchoring the default network, were specific to numeracy, but not literacy. Differences in the nature of the two WIAT tests (i.e., more intermediate steps involved in Numerical Operation) could influence our iFC findings, although testing this possibility is beyond the scope of the current study. Alternatively, visuospatial working memory may explain the observed iFC differences between literacy and numeracy, in considering cognitive demands that can be more or uniquely required for numeracy relative to literacy^67,68,73^. This appears to be a plausible explanation, given the well-demonstrated links between working memory and default network networks^74–76^, particularly the negative correlations between these two networks during the maintenance phase of visuospatial working memory^77^. However, future work using more targeted tasks would be required to provide direct support for this hypothesis.

Another possible explanation can be offered by Kelly *et al*.^78^; the effect of the negative connectivity-behavior relationship, specifically involving iFC between the task-positive network and the task-negative default network, was more evident for a higher demand process (i.e., the incongruent condition in a flanker task) than a lower demand one (i.e., the congruent condition). From this finding, we can infer that the observed pattern specific to numeracy can be attributed to a possibility that “Numerical Operations” places higher cognitive demands relative to “Word Reading”. This assumption can be supported by results of our secondary analysis, focusing on the discrepancy in the standard scores between the two WIAT sub-tests. Specifically, individuals with “Superior Numerical” (Numerical Operations > Word Reading) tended to have weaker positive (or stronger negative) iFC between R.MFG and each default network region. In these individuals, cognitive demands required for the performance may be lower for Numerical Operations relative to Word Reading. Importantly, this finding is consistent with negative connectivity-behavior relationships observed for the same connections and Numerical Operations scores (i.e., individuals with higher numeracy scores tended to have weaker positive/stronger negative iFC). Future studies are necessary to confirm whether the observed iFC differences between literacy and numeracy are present even when well-matched tasks/tests for these two cognitive domains are used.

In addition to the default network circuitry, the R.MFG connections with key regions in the salience network (dACC, Insula), which detect/select stimuli deserving of our attention^43,79^, were positively associated with numeracy. These regions are often co-activated during a wide range of cognitive tasks^80–83^, including numerical processing^84,85^. In particular, the anterior part of the insula has become a focus of exploration beyond its known functions, such as perception, emotion, and self-awareness^86,87^. For example, a meta-analytic study focusing on the insula revealed that the dorsal anterior insula is consistently involved in cognitive processes^88^, consistent with R-fMRI parcellation results^89^. More relevant to our iFC findings, Superkar and Menon (2012)^90^ have used multivariate approaches, demonstrating a causal interaction between R.Insula and the right dorsolateral prefrontal cortex during arithmetic problem solving in both children and adults. Together with previous task-evoked findings, our iFC results indicate that the R.MFG connections with the salience network, particularly R.Insula, support numeracy, most likely due to its functions in attentional and cognitive control that enable efficient learning^42^, particularly for cognitively challenging tasks^83^, like “Numerical Operations”. Of note, we observed that only the R.MFG connection with R.Insula (a “numeracy positive connection”) exhibited a significant negative correlation with R.MRG connections with default network regions (“numeracy negative connections”). This can be potentially explained by R.Insula’s role in switching the two opponent systems − deactivating the task-negative default network and activating the task-positive central executive network^91^.

The R.MFG connections with dACC and Cereb also each positively correlated with literacy. These iFC profiles may be common neural mechanisms underlying literacy and numeracy, given that each process – online-monitoring of performance^92^ and automatization^93,94^ – as a key function of dACC and Cereb, respectively, is required for skilled learning. Given that dorsolateral prefrontal connections with the anterior cingulate cortex^95^ and cerebellum^96^ are associated with executive function of attention, it may be that prefrontal (i.e., R.MFG) connections with dACC and Cereb can be common neural correlates underlying any two or more competencies/tasks which require attention. As shown by behavioral studies, attention is a common cognitive component involved in literacy and numeracy^21,22^. As discussed above, the Numerical Operations may place higher cognitive demands relative to the Word Reading. This, together with a slightly wider inter-subject variability in the Numerical Operations (SD = 15.90, relative to 11.19 for the Word Reading), may have contributed to the detection of significant connectivity-behavior relationships (R.MFG connections with dACC and Cereb) only for numeracy. Yet, a conclusive interpretation to posit these iFC profiles as common mechanisms for literacy and numeracy awaits further research with a larger sample size, particularly including adequate number of comorbid individuals in these two competencies.

Similar to a set of the R.MFG connections specific to numeracy, we also found iFC profiles specific to literacy. Literacy was positively associated with L.MFG connections with an adjacent prefrontal region in the left hemisphere (i.e., L.dMFG), as well as with the homotopic MFG region (i.e., R.MFG-lit). These results indicate that closer functional coupling within the dorsolateral prefrontal cortex is optimal for literacy performance. When considering each L.MFG connection, the observed intra-hemispheric prefrontal iFC emphasizes the importance of left-lateralization in literacy, which is consistent with prior iFC findings^25,27,28^, structural connectivity findings^97^, and task-evoked activation findings^7,57^. In contrast, a contribution of inter-hemispheric prefrontal iFC to literacy has been rarely reported, except for a pilot R-fMRI study (only 5 dyslexic children) that showed reduced inter-hemispheric iFC within the inferior frontal gyrus in dyslexics^98^. Given that the integrity of inter-hemispheric prefrontal iFC is associated with efficient attentional processing^99^, and also that attentional mechanisms play a crucial role in reading fluency^100^, our finding indicates that successful literacy relies on the crosstalk in the dorsolateral prefrontal cortex associated with attention^101^.

Finally, the current study found no iFC of some brain regions (e.g., the left fusiform gyrus) that have been detected/reported by previous fMRI studies on literacy^7,102^ and those on numeracy^3,7^. This may be due to fundamental differences in methodological approaches; the current study used a task-independent R-fMRI approach, focusing on the exploratory MDMR (followed by SCA using the MDMR results), whereas the majority of previous studies used task-evoked fMRI approaches (e.g., phonological judgement for literacy^103^; numerical magnitude^54^ and simple arithmetic operations^24^ for numeracy). Even for R-fMRI studies on these two competencies, including our prior work^25,28,104^, many of them examined iFC patterns of seeds selected from set of regions showing task-evoked activations^102^ (e.g., positive iFC between the left fusiform gyrus seed selected from a meta-analysis^102^ and the left inferior frontal gyrus for word reading^28^). Importantly, the current study attempted to minimize biases attached to seed selection^105^ and to identify potentially important iFC patterns of brain regions in literacy and numeracy, beyond regions that have been detected by previous task-evoked fMRI studies.

In conclusion, MDMR, an exploratory multivariate analysis, highlights the L.MFG and R.MFG nodes within the multiple demand system for literacy and numeracy, respectively. Post-hoc SCA results further reveal differential contributions of MFG connections to each of the two competencies, consistent with a recent review of neuroimaging studies^7^, and also indicate potentially common IFC profiles to both competencies that require attentional demands. One of the most striking and novel findings from the present work is the involvement of the default network circuitry (i.e., the R.MFG connections with vmPFC, ITG, LPC, and PCC/Pre) in numeracy, though largely overlooked or understudied in the literature. These results indicate a beneficial role of stronger negative (or weaker positive) connectivity between a task-positive prefrontal region (i.e., R.MFG) and task-negative default network regions for successful numeracy. Future work using more targeted tasks would be required to identify factors that contribute to distinct and common iFC profiles in literacy and numeracy. Taken together, our iFC findings provide novel insights into neural bases of literacy, numeracy, and impairments in each competency or both.

## Methods

### Participants

The present study made use of data from the ongoing NKI-Rockland Sample (NKI-RS) initiative^26^. The NKI-RS is a community-ascertained multimodal-imaging sample of individuals between the ages of 6.0 and 85.0. The NKI-RS study has been carried out with the approval of the institutional review board at the Nathan Kline Institute for Psychiatric Research. Prior to participation, written informed consent was obtained from all participants in accordance with local institutional review board oversight. All experimental methods were carried out in accordance with the relevant guidelines and regulations of the NKI.

Participants were selected from the data available at the time of analysis (n = 521). A total of seventy healthy adults were included in further statistical analyses, after applying the following inclusion criteria: 1) age: 20.0–49.0 years old, 2) intelligence quotient (IQ) higher than 80 on each of all three measures (verbal, perceptual, and full scale) obtained by the WASI^106^, 3) English as the first or dominant language used at/outside home, 4) right handedness as assessed by the Edinburgh Handedness Inventory^107^, 5) absence of any current medical conditions (e.g., hypothyroidism, diabetes), or accompanying medications (e.g., Levothyroxine, Metformin), that could influence the BOLD signal^108,109^, 6) absence of current or previous diagnoses of DSM-IV-TR Axis-I psychiatric disorders (based on the Structured Clinical Interview for DSM-IV)^110^, 7) absence of clinically elevated scores (>70) on the Conners’ Adult ADHD Rating Scales (CAARS)^111^, 8) mean frame-wise displacement (FD)^112^ during the R-fMRI scan < 0.2 mm to minimize motion artifacts, and 9) structural and R-fMRI scans without artifacts detectable by visual data inspection.

### Measures of Literacy and Numeracy

Literacy and numeracy competencies were assessed using two subtests of the Wechsler Individual Achievement Test III (WIAT-III), “Word Reading” and “Numerical Operations”, respectively. The WIAT Word Reading measures accuracy of reading single words that increase in difficulty. For the WIAT Numerical Operations, which is designed to estimate written arithmetic calculation abilities, questions range from basic counting to more complex operations including multiplication, fraction, integers, geometry, algebra, and calculus. For both subtests, questions were visually presented, and correct responses were scored until a participant made 4 consecutive “0” scores. In addition to our primary aim to link iFC to these two competencies, we also examined the prevalence of potential LD with each competency (and impairments with both competencies) in our sample. Note that “potential LD” or “impairment” in the current study was defined as having a standard score lower than 85 (i.e., −1SD).

### MRI Data Acquisition

All MRI data were collected using a Siemens Trio 3.0 Tesla scanner located at the Center for Biomedical Imaging and Neuromodulation (CBIN) at the NKI, as part of the standard NKI-RS scan session. Each participant completed a 10-minute R-fMRI scan optimized for temporal resolution, which was comprised of 900 contiguous whole-brain functional volumes acquired using a multiband echo-planar imaging (EPI) sequence (effective TE = 30 ms; TR = 645 ms; flip angle = 60°; 40 slices; voxel-size = 3 × 3 × 3 mm; field of view = 222 mm). During the scan, participants were instructed to remain still and keep their eyes open. A high-resolution T1-weighted anatomical image was also acquired using a magnetization prepared gradient echo sequence (MPRAGE, TE = 2.52 ms; TR = 1900 ms; TI = 900 ms; flip angle = 9°; 176 slices; acquisition voxel size = 1.0 × 1.0 × 1.0 mm; field of view = 250 mm).

### MRI Data Preprocessing

MRI data preprocessing was carried out using the Configurable Pipeline for the Analysis of Connectomes (CPAC version 0.3.9.1 http://fcp-indi.github.io/docs/user/index.html). Our fMRI preprocessing included the following steps: realignment to the mean EPI image to correct for motion, grand mean-based intensity normalization (all volumes scaled by a factor of 10,000), nuisance regression, spatial normalization, temporal band-pass filtering (0.01–0.1 Hz), and spatial smoothing.

Nuisance regression was performed to control for the effects of head motion and to reduce the influence of signals of no interest. The regression model included linear and quadratic trends, the Friston-24 motion parameters (6 head motion, their values from one time point before, and the 12 corresponding squared items)^113^, and the signals of five principal components derived from noise regions of interest (e.g., white matter, cerebral spinal fluid) using a component-based noise correction method (CompCor)^114^.

Spatial normalization included the following steps: (1) anatomical-to-standard registration using Advanced Normalization Tools (ANTs, http://www.picsl.upenn.edu/ANTS)^115^, which has been demonstrated to have superior performance compared to other commonly used registration algorithms^116,117^; (2) functional-to-anatomical registration using FLIRT with a 6-degrees of freedom linear transformation, which was further refined using the Boundary-based Registration implemented in FSL^118^; and (3) functional-to-standard registration by applying the transformation matrices obtained from step (1) and (2) using ANTs. Spatial smoothing was performed using a Gaussian kernel (Full width at half maximum = 6 mm).

### Multivariate Distance Matrix Regression (MDMR)

We applied the MDMR framework, as an exploratory analysis tool, to the preprocessed R-fMRI data^35^. MDMR is designed to identify brain regions whose inter-individual variation in whole-brain iFC profiles is related to inter-individual variation in one or more phenotypic variables. MDMR was applied using the “Connectir” package in R (http://czarrar.github.io/connectir) on resampled, 3 mm^3^ isotropic voxels.

MDMR was performed on a voxel-by-voxel basis for the following three steps: 1) Pearson’s correlations were computed between the time series of the target voxel and that of all other voxels to generate the whole-brain iFC for the target voxel, for each participant; 2) A between-participant distance matrix was computed, in which each entry is the distance between the iFC maps obtained for the target voxel in two different participants. Note that “distance” was defined as √(2*(1−r)), where r is the spatial correlation of the iFC maps obtained at the target voxel in two different participants; 3) A pseudo-F statistic was computed to provide mathematical evaluation of the relationship between the variability in the distance matrix computed in Step 2 and the variable of interest (i.e., literacy, numeracy). Voxel-wise significance of the pseudo-F statistic was determined via estimation of the null distribution with random permutation (n = 10,000). Thus, the pseudo-F value at each voxel tells us whether the iFC profiles for that voxel varied among individuals as a function of the phenotypic variable (i.e., literacy, numeracy). In other words, the phonetic variable of interest (e.g., Word Reading) was regressed onto the distance matrix, to test if that variable could explain the variability in the distance matrix. Note that all the computations were constrained to a study-specific group mask, including only voxels present across all participants and contained in a 25% probability gray-matter MNI mask.

There were two statistical models employed for MDMR – one with literacy (Word Reading) included as the covariate of interest, and another with numeracy (Numerical Operations). Both models included the following covariates of non-interest; age, sex, handedness, mean FD, and global connectivity (calculated using @compute_gcor, an AFNI command). Of note, IQ was not entered as a covariate of non-interest, given that controlling for the effect of IQ could potentially produce overcorrected, anomalous, and counterintuitive findings about neurocognitive functions^119^. Crucially, in response to recent discussions about cluster-extent thresholding^120,121^, we corrected for multiple comparisons using cluster-based extent thresholding, with a height (i.e., cluster-forming) threshold of *Z* > 3.1 (corresponding to *p < *0.001) and cluster-extent probability of *p* < 0.05 (FWER corrected).

### Seed-based correlation analyses (SCA)

MDMR does not specify the nature or direction of the connectivity-behavior relationship, and thus we performed post-hoc SCA using regions detected by MDMR. The average time-series across all voxels within each regions of interest were extracted and correlated with all voxels within the study-specific group mask using Pearson’s correlation. Correlation values were transformed to Fisher Z scores to provide a whole-brain iFC map. The same group model used for MDMR was applied to subsequent group-level SCA. The resultant iFC maps were corrected for multiple comparisons using GRF (*Z* > 3.1; *p* < 0.05).

## Electronic supplementary material

Supplementary Figure 1
