# Supplementary material for: Differential contributions of the middle frontal gyrus functional connectivity to literacy and numeracy
Source: Sci Rep. 2017 Dec 13;7:17548. doi: 10.1038/s41598-017-17702-6 (PMC5727510; doi:10.1038/s41598-017-17702-6)
Supplement: Supplementary file 1 — Supplementary Figure 1 [file 41598_2017_17702_MOESM1_ESM.pdf]

TITLE

Differential contributions of the middle frontal gyrus functional connectivity to literacy and numeracy

AUTHORS

Maki S. Koyama[1,2], David O'Connor[3], Zarrar Shehzad[4], Michael P. Milham[1,3]

1. Nathan Kline Institute for Psychiatric Research (NY, USA)
2. Haskins Laboratories (CT, USA)
3. Child Mind Institute (NY, USA)
4. Yale University, Department of Psychology (CT, USA)

CORRESPONDING AUTHORS

Maki S. Koyama makisophiakoyama@gmail.com

Michael P. Milham michael.milham@childmind.org

SUPPLEMENTARY MATERIAL

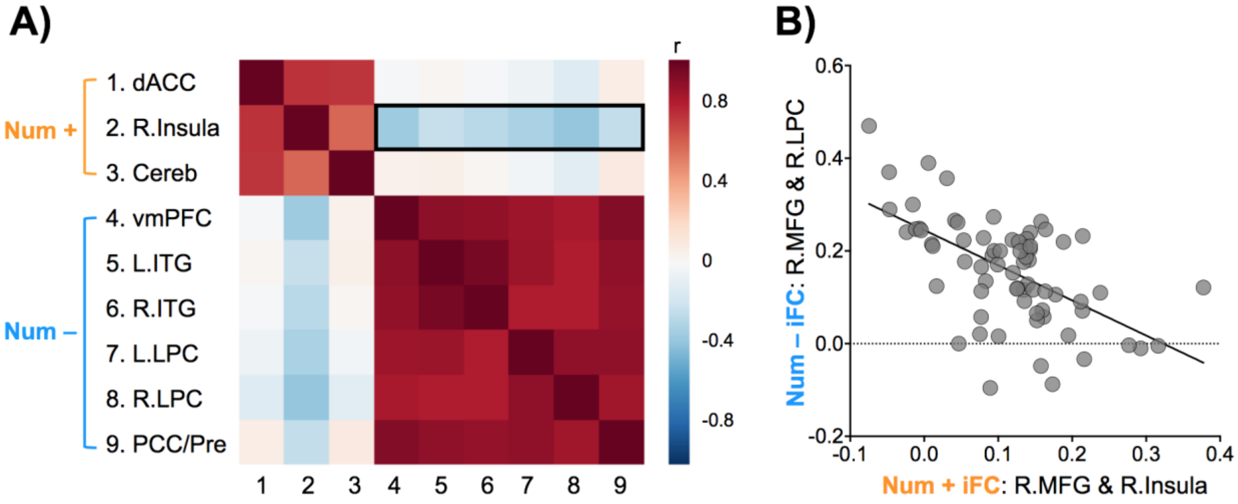

Supplementary Figure 1. Relationships between the right middle frontal gyrus (R.MFG) connections that are positively and negatively correlated with numeracy. (A) A correlation matrix highlights that connectivity strength between R.MFG and right insula (R.Insula) – a numeracy positive (Num +) connection – had a significant negative correlation with each of numeracy negative (Num –) connections of R.MFG with default network regions (the black-lined rectangle). This indicates functional segregation between the two opponent network systems. Additionally, R.MRG connections were strongly coupled/integrated (i.e., positive correlations) within each of the "Num + connections" and "Num – connections". **(B)** A scatter plot depicts a significant negative association between a "Num + connection" and "Num – connections". Specifically, R.MFG iFC with R.Insula was negatively correlated (*r* = -0.58) with R.MFG iFC with right lateral parietal cortex (R.LPC), a region within the default network. This pattern was observed for other default network regions, that is, individuals with stronger positive connectivity strength in the "Num + connection" between R.MFG and R.Insula tended to have weaker positive (or stronger negative) connectivity strength in "Num – connections" between R.MFG and the default network. dACC = dorsal anterior cingulate cortex, Cereb = cerebellum, vmPFC = ventromedial prefrontal cortex, ITG = interior temporal gyrus, LPC = lateral parietal cortex, PCC/Pre = posterior cingulate cortex/precuneus, L=Left, R=Right
